# Irritable bowel syndrome (IBS) among Lebanese adults: unidentified IBS and associated factors

**DOI:** 10.1186/s12889-023-16543-5

**Published:** 2023-08-22

**Authors:** Gabriella Yazbeck, Diana Malaeb, Hamid Shaaban, Abir Sarray El Dine, Souheil Hallit, Rabih Hallit

**Affiliations:** 1 School of Medicine and Medical Sciences, Holy Spirit University of Kaslik, P.O. Box 446, Jounieh, Lebanon; 2 College of Pharmacy, Medical Gulf University, Ajman, United Arab Emirates; 3 New York Medical College, New York, USA; 4 Department of Infectious Disease, Saint Michael’s Medical Center, Newark, NJ USA; 5 Department of Biomedical Sciences, School of Arts and Sciences, Lebanese International University, Beirut, Lebanon; 6 Applied Science Research Center, Applied Science Private University, Amman, Jordan; 7 Research Department, Psychiatric Hospital of the Cross, Jal Eddib, Lebanon; 8 Department of Infectious Disease, Bellevue Medical Center, Mansourieh, Lebanon; 9 Department of Infectious Disease, Notre Dame des Secours University Hospital Center, Street 93, Byblos, Postal Code 3 Lebanon

**Keywords:** Irritable bowel syndrome, Unidentified, Psychological disorders, Lebanon, Factors

## Abstract

**Background:**

Irritable bowel syndrome (IBS) is one of the most frequent functional gastrointestinal disorders, but the condition is still underdiagnosed. The high of rate of unidentified IBS by patients can be related to different factors. The aim of this study is to assess the rate of unidentified IBS among Lebanese adults and investigate the role of socio-demographic factors, anxiety, depression, insomnia and eating attitudes on IBS diagnosis.

**Methods:**

A cross-sectional study was conducted among Lebanese adults older than 18 years between June 2022 and December 2022, using a self-reporting questionnaire distributed via social media.

**Results:**

A total of 425 participants was enrolled in the study with around 184 (46.8%) having a possible unidentified IBS. Higher psychological distress (aOR = 1.07) and insomnia severity (aOR = 1.08) were significantly associated with higher odds of having possible unidentified IBS whereas a higher household crowding index (aOR = 0.67) was significantly associated with lower odds of having possible IBS. The correlation of eating attitudes with cigarette smoking (aOR = 1.33; *p* = .025; 95% CI 1.04; 1.70) and insomnia severity with cigarette smoking (aOR = .89; *p* = .023; 95% CI .80; .98) were significantly associated with the presence of possible IBS. In nonsmokers, higher psychological distress (aOR = 1.07) and insomnia severity (aOR = 1.10) were significantly associated with higher odds of having possible IBS. In smokers, higher BMI (aOR = .78) was significantly associated with lower odds of having possible IBS, whereas higher eating attitudes scores (more inappropriate eating) (aOR = 1.40) were significantly associated with higher odds of having possible IBS.

**Conclusion:**

The study highlighted the implication of raising awareness about IBS among the Lebanese population to promote early diagnosis and minimize the rate of unidentified IBS by patients. Initiation of appropriate treatment plans, tailored symptomatic management approach, and diet programs should be highly encouraged.

## Introduction

Irritable bowel syndrome is one of the most frequent functional gastrointestinal disorders characterized by a cluster of symptoms including altered bowel movements, associated with abdominal pain without any detectable biochemical, structural abnormalities or underlying damages to the intestines [1]*.* The etiology of IBS is still unknown, but certain risk factors have been linked to its pathogenesis [2]. Furthermore, there are no diagnostic tests or biomarkers for IBS, and it can only be identified by symptom-based assessment guidelines [3]*.* According to previous literature, the prevalence of IBS ranges from 1.1% to 45%, based on population studies from nations throughout the world, with a pooled prevalence of 11.2% [4]. An observational cross-sectional study focusing on bank employees in Lebanon, aged between eighteen and sixty five years old, and another one conducted among Lebanese university students revealed a prevalence of 20.1% and 20% of IBS respectively according to Rome III criteria [5, 6].

IBS was found to be underdiagnosed among American athletes where only 2.8% were diagnosed from a total of 9.8% suffering from IBS [7]. A study conducted in Hong Kong showed a significant underdiagnosis of IBS with only 21% of participants having IBS [8]. According to another study conducted on patients seeking healthcare services, underdiagnosis of IBS reached 66% with only 33% being previously diagnosed of having IBS [9]. Unidentified IBS may also be prevalent among Lebanese adults.

According to previous studies, IBS is influenced by sociodemographic factors such as age, psychiatric disorders, sleep disturbances and inappropriate eating attitudes. A meta-analysis showed a higher prevalence of IBS among women [4]; which coincides with the results done in Lebanon [5, 6]. However two studies conducted in Singapore and Taiwan didn’t show difference regarding the prevalence of IBS between males and females [10, 11].

IBS has been found to be higher among single and divorced individuals [12, 13]. The association between IBS and educational level is controversial; some studies showed that lower levels of education are associated with IBS [14], while other studies showed the opposite [12, 15]. However, some studies showed no association between education and IBS [16, 17]. Results are also controversial regarding the association between body mass index (BMI) and IBS [13, 15, 18–21]. Hiowever, most of the evidence points that a strong association exists between obesity and IBS [22, 23]. Finally, IBS has been shown to be more prevalent among Lebanese university students with low or no regular physical activity [6]; similar results were found in international studies [24–26].

Alcohol consumption was found to be associated with IBS in many published studies [5, 24, 27]. Prolonged alcohol intake can alter the permeability and the motility of the gastrointestinal tract by interfering with the intestinal smooth muscle activity [28]. Alcohol can also trigger and exacerbate the symptoms of IBS [29, 30]. However, other studies did not show an association between alcohol drinking and IBS [31, 32]. Results concerning the association of IBS and cigarette smoking were controversial according to different studies across the world. A meta-analysis and a Lebanese study conducted among bank employees in addition to a study conducted in Iran did not show a significant association between cigarette smoking and IBS [5, 33–35]. A significant association between IBS and non-cigarette smoking was showed in a study conducted in Pakistan in the year 2018 [36], while participants who regularly smoke cigarettes reported to have higher prevalence of IBS among Saudi university students [26]. The literature was not conclusive concerning the association between waterpipe smoking and IBS. When compared to non-waterpipe users, waterpipe smokers had considerably higher IBS [5]. However, no association between smoking waterpipe and IBS was found in Iran [33].

IBS and psychiatric disorders are known to be strongly associated. According to two meta-analysis, IBS patients exhibited significantly greater levels of anxiety and depression than controls [37, 38]. Furthermore, more severe and more frequent symptoms related to depression were assessed among IBS patients according to another meta-analysis [39]. Moreover, a cohort study revealed that IBS candidates are more prone than controls to develop anxiety, depression and sleep disturbances [40]. These findings can be explained by the fact that many brain nuclei that regulate normal gastrointestinal mechanisms also control physiologic, emotional, and fear-conditioning response to danger [41]. A reciprocal relationship is reported to be present between the brain and the gut in the pathogenesis of IBS with the interference of the autonomic nervous system [42]. A meta-analysis conducted in 2018 has shown a higher prevalence of sleep disturbances among IBS patients [43]. Another study conducted in 2021 showed the presence of insomnia and/or hypersomnelence in 73% of the subjects suffering from IBS while it showed a rate of 37% of insomnia and/or hypersomnolence among the controls [44]. According to a study conducted among Saudi undergraduate students, individuals with self-reported sleep disturbances had higher prevalence of IBS [26].

Eating disorders were found to be very common among IBS patients. A cross-sectional study conducted via online platforms among IBS patients concluded an association between IBS and eating attitudes [45]. A case–control study found that eating disorders were more prevalent among the case group which included participants having IBS in comparison with the control group [46]. Another study assessing the prevalence of IBS among patients having a history of eating disorders or current eating disorders resulted in 64% having IBS [47].

The literature lacks information related to the unidentification of IBS among Lebanese adults as well as the potential associated factors especially the inappropriate eating attitudes. Lebanon is going through a hard period due to economic problems that can negatively affect the prevalence of IBS. Studies conducted among the Lebanese population during the economic and political crisis found a high rate of food insecurity [48, 49] which consequently leads to increased inappropriate eating attitudes. Another study conducted among Lebanese adults during the COVID-19 pandemic that coexisted with the economic crisis showed a high prevalence of depression, anxiety and stress [50]. In addition, the economic difficulties can decrease the ability of Lebanese people to afford medical care. These different factors can play a role in affecting the rate of unidentified IBS. Previous Lebanese studies regarding IBS were conducted before the beginning of the socioeconomic and political crisis since 2020 with no recent studies. The aim of this study is to assess the rate of unidentified IBS among Lebanese adults and investigate the role of socio-demographic factors, anxiety, depression, insomnia, and eating attitudes, on IBS diagnosis during the Lebanese crisis.

## Methods

### Study design

A cross-sectional study was conducted in Lebanon between June and December 2022 by using the snowball sampling technique. The digital questionnaire was built using Google Forms and distributed online via social media platforms. The research team approached people they know, who were asked to forward the link to other friends, coworkers and family members they know. We included all Lebanese adults aged more than 18 years old and excluded participants with a previous physician-diagnosed of IBS and those who refused to participate by filling out the questionnaire.

### Minimal sample size calculation

Minimal Sample Size calculation was done using the G-Power software with a R^2^ deviation from zero of 0.05, a type I error of 5%, a power of 80% and 15 predictors to be entered in the final model. The required minimal sample of participants was 371.

### Questionnaire

A validated, confidential, anonymous and self-reporting questionnaire in Arabic was used; it included validated scales and scoring systems and other questions covering sociodemographic details (age, sex, weight, height, marital status, education (dichotomized in secondary education or less and university education) cigarette smoking, water pipe smoking, alcohol consumption, and physical activity) as well as other questions about the house crowding index [35], and previous diagnosis of IBS by a physician. The following scales were used in our questionnaire:

#### Rome IV diagnostic questionnaire (for adults)

This questionnaire was used for the diagnosis of irritable bowel syndrome [51]. Before starting the data collection, the Rome Foundation granted permission to use the Rome IV diagnostic questionnaire with an Arabic translation. To be diagnosed with possible IBS, the participant must fulfill the following criteria for the past 3 months: (1) Recurrent abdominal pain at least weekly, (2) Pain is associated with two or more of the following criteria: (a) related to defecation at least 30% of occasions, (b) associated with a change in frequency of stool at least 30% of occasions, (c) associated with a change in form (appearance) of stool at least 30% of occasions and (3) having symptom onset at least 6 months prior to diagnosis (Cronbach’s alpha in this study = 0.74).

#### Depression Anxiety Stress Scale (DASS-8)

This scale is a shorter version of the Depression Anxiety Stress Scale, DASS-21 which is composed of 21 questions, both scales are validated in the Arabic language [52, 53]. This questionnaire investigates three components: Depression (3 items (e.g., “felt down hearted and blue”)), Anxiety (3 items (e.g., “felt scared without reason”)) and Stress (2 items (e.g., “was using a lot of my mental energy”)); each item is scored from 0 (“did not apply to me at all”) to 3 (“applied to me very much or most of the time”), with a minimum total score of 0 and a maximum total score of 24 based on the perceived severity of symptoms; with higher scores reflecting higher risk of mental distress [52, 53] (Cronbach’s alpha in this study = 0.91).

#### Insomnia Severity Index (7 questions)

We investigated insomnia using the Arabic version of the Insomnia Severity Index including 7 items. Insomnia and sleeping related disorders were assessed according to the last two weeks, with each item scored from 0 to 4 according to the Likert-type scale and based on the perception of the intensity of their symptoms. The total score ranges from 0 to 28 with a higher score indicating worse insomnia severity [54]. The Arabic version of this index was validated in Lebanon with a good reliability [55] (Cronbach’s alpha in this study = 0.81).

#### Eating Attitudes Test (EAT-7)

A shorter Arabic version of the scale was validated in Lebanon, the EAT-7, with a good reliability and accuracy [56]. Responders designated how frequently each component is related to them. Each item is scored from 0 to 3 (0: “never”, “rarely” or “sometimes”, 1: “often”, 2: “usually”, 3: “always”) (Cronbach’s alpha in this study = 0.73). 

### Statistical analysis

The SPSS v.25 software was used for the statistical analysis. The Chi-square test was used to compare two categorical variables, whereas the Student t test was used to compare two means. A logistic regression, using the ENTER method and taking the presence vs absence of IBS according to the ROME IV criteria as the dependent variable, was conducted. All variables that showed a *p* < 0.25 in the bivariate analysis were taken as independent variables in the model. The interaction eating attitudes/insomnia severity by cigarette smoking was tested; a stratification analysis according to the smoking status was deemed necessary if the interaction(s) was significant. *P* < 0.05 was deemed statistically significant.

## Results

The questionnaire was filled by a total of 425 participants. The mean age was 24.53 ± 8.57 years, with 76.8% females. According to the ROME IV criteria, the results showed that 184 (46.8%) might have a possible unidentification of IBS. Other characteristics of the sample can be found in Table 1.

**Table 1:** Sociodemographic and other characteristics of the participants (*n* = 393)

	**n (%)**
Sex	
Male	91 (23.2%)
Female	302 (76.8%)
Marital status	
Single	328 (83.5%)
Married	65 (16.5%)
Education	
Secondary or less	44 (11.2%)
University	349 (88.8%)
Undiagnosed IBS according to ROME IV criteria	
No	209 (53.2%)
Yes	184 (46.8%)
	**Mean ± SD**
Age, years	24.53 ± 8.57
Body Mass Index (kg/m2)	23.11 ± 4.55
Household crowding index (person/room)	1.33 ± .61
Physical activity	21.24 ± 19.79
Eating attitudes	1.76 ± 2.97
Psychological distress	9.22 ± 7.13
Insomnia severity	8.46 ± 5.48

### Bivariate analysis

The results of the bivariate analysis are shown in Table 2. A higher percentage of females had possible IBS according to the ROME IV criteria compared to males. Moreover, higher mean eating attitudes, psychological distress and insomnia severity scores, as well as lower mean BMI, and physical activity were significantly found in participants who had possible IBS.

**Table 2:** Bivariate analysis of factors associated with the presence of IBS according to the ROME IV criteria

Variable	Absence of IBS (*N* = 209; 53.2%)	Presence of IBS (*N* = 184; 46.8%)	X^2^ / t	*P*
Sex			13.99	** < .001**
Male	64 (70.3%)	27 (29.7%)		
Female	145 (48.0%)	157 (52.0%)		
Marital status			.87	.350
Single	171 (52.1%)	157 (47.9%)		
Married	38 (58.5%)	27 (41.5%)		
Education			3.22	.073
Secondary or less	29 (65.9%)	15 (34.1%)		
University	180 (51.6%)	169 (48.4%)		
Cigarette smoking			2.16	.142
No	173 (51.6%)	162 (48.4%)		
Yes	36 (62.1%)	22 (37.9%)		
Waterpipe smoking			.18	.676
No	136 (54.0%)	116 (46.0%)		
Yes	73 (51.8%)	68 (48.2%)		
Alcohol drinking			.18	.670
No	187 (52.8%)	167 (47.2%)		
Yes	22 (56.4%)	17 (43.6%)		
Age	25.30 ± 9.58	23.66 ± 7.18	1.93	.054
Body mass index	23.62 ± 4.76	22.53 ± 4.25	2.39	**.017**
Household crowding index	1.37 ± .64	1.28 ± .57	1.53	.127
Physical activity	23.77 ± 20.73	18.36 ± 18.30	2.74	**.006**
Eating attitudes	1.43 ± 2.50	2.13 ± 3.39	2.30	**.022**
Psychological distress	7.07 ± 6.27	11.67 ± 7.28	6.67	** < .001**
Insomnia severity	7.03 ± 5.05	10.08 ± 5.51	5.73	** < .001**

Numbers in bold indicate significant *p* values

### Multivariable analysis

The result of a logistic regression taking the presence vs absence of IBS according to the ROME IV criteria as the dependent variable, showed that higher psychological distress (aOR = 1.07) and insomnia severity (aOR = 1.08) were significantly associated with higher odds of having possible IBS. Moreover, a higher household crowding index (aOR = 0.67) was significantly associated with lower odds of having possible IBS (Table 3).

**Table 3:** Multivariable analysis: Logistic regression (using the ENTER method) taking the presence vs absence of IBS according to the ROME IV criteria as the dependent variable (*R*^*2*^ = .218)

Variable	*P*	Aor	95% CI
Sex (females vs males*)	.193	1.46	.83; 2.59
Education (university vs secondary or less*)	.432	1.37	.63; 2.97
Cigarette smoking (yes vs no*)	.312	.704	.36; 1.39
Age	.505	.99	.96; 1.02
Body mass index	.184	.97	.92; 1.02
Household crowding index	**.038**	.67	.46; .98
Physical activity	.077	.99	.98; 1.00
Eating attitudes	.502	1.03	.95; 1.11
Psychological distress	**.001**	1.07	1.03; 1.11
Insomnia severity	**.003**	1.08	1.03; 1.13

Numbers in bold indicate significant *p* values

^*^indicates the reference group

### Moderation analysis

The interaction eating attitudes by cigarette smoking (aOR = 1.33; *p* = 0.025; 95% CI 1.04; 1.70) and insomnia severity by cigarette smoking (aOR = 0.89; *p* = 0.023; 95% CI 0.80; 0.98) were significantly associated with the presence of possible IBS according to the ROME IV criteria. Consequently, a stratification analysis was according based on the smoking status. In nonsmokers, higher psychological distress (aOR = 1.07) and insomnia severity (aOR = 1.10) were significantly associated with higher odds of having possible IBS. In smokers, higher BMI (aOR = 0.78) was significantly associated with lower odds of having possible IBS, whereas higher eating attitudes scores (more inappropriate eating) (aOR = 1.40) were significantly associated with higher odds of having possible IBS (Table 4).

**Table 4:** Stratification analysis according to the smoking status: Logistic regressions (using the ENTER method) taking the presence vs absence of IBS according to the ROME IV criteria as the dependent variable

	**Nonsmokers (*R***^***2***^** = .225)**			**Smokers (*R***^***2***^** = .423)**		
**Variable**	***P***	**aOR**	**95% CI**	***P***	**aOR**	**95% CI**
Sex (females vs males*)	.750	1.11	.59; 2.10	.222	2.73	.55; 13.64
Education (university vs secondary or less*)	.648	1.24	.50; 3.10	.101	6.46	.69; 60.14
Age	.570	.99	.96; 1.03	.680	1.02	.94; 1.10
Body mass index	.688	.99	.93; 1.05	**.011**	.78	.65; .95
Household crowding index	.058	.67	.45; 1.01	.67	1.41	.29; 6.78
Physical activity	.098	.99	.98; 1.00	.35	.98	.95; 1.02
Eating attitudes	.453	.97	.89; 1.06	**.024**	1.40	1.05; 1.89
Psychological distress	**.001**	1.07	1.03; 1.12	.176	1.09	.96; 1.25
Insomnia severity	** < .001**	1.10	1.05; 1.17	.203	.90	.76; 1.06

Numbers in bold indicate significant *p* values

*aOR* Adjusted Odds Ratio

^*^indicates the reference group

## Discussion

Our objectives in this study were to asses the possible unidentification of IBS among Lebanese adults, and to investigate possible associated factors: socio-demographic factors, anxiety, depression, insomnia, and inappropriate eating attitudes, on IBS diagnosis. We found a very high rate of unidentified of IBS among the Lebanese adult population, as well as a significant association between IBS and psychiatric disorders, and insominia. We also found a moderating effect of cigarette smoking, in nonsmokers, higher psychological distress and insomnia severity were significantly associated with higher odds of having possible IBS. In smokers, higher BMI was significantly associated with lower odds of having possible IBS, whereas higher eating attitudes scores (more inappropriate eating) were significantly associated with higher odds of having possible IBS.

### Unidentified IBS

In this study, the rate of possible unidentified IBS was 46.8% using the Rome IV criteria. Previous studies conducted in Lebanon among bank employees and university students based on the Rome III criteria showed a significantly lower prevalence of IBS [5, 6]. Higher rates of possible unidentification of IBS were also reported in our study in comparison with other studies conducted in other countries. A population-based study conducted in the United States, Canada and the United Kingdom based on a self-assessment questionnaire using the Rome IV Diagnostic Questionnaire and Rome III irritable bowel syndrome (IBS), showed a prevalence of IBS ranging from 4.4% to 4.8% [57]. Another study conducted in Poland, among participants of the 2018 Woodstock Rock Festival using questionnaires based on the Rome IV criteria showed a rate of 11% of IBS [58]. A cross-sectional study conducted in Riadh in 3 out-patients clinics using self-assessment questionnaires distributed via social media and based on the Rome IV criteria showed a prevalence of 7.9% of IBS [59]. IBS prevalence was reported to be 7.4% according to a nationwide study conducted in the USA by using an online survey based on the Rome IV criteria during the peak of the coronavirus first wave [60]. The increasing prevalence of IBS among Lebanese adults may be due to psychiatric disorders like anxiety, depression and sleep disturbances. Lebanon has been going through a hard period due to socioeconomic and food insecurities as well as the political problems,but the majority of our participants have a university degree and live in a household with less than two persons per room which indicated that socioeconomic and food insecurities may not be associated with high rates of IBS in our study.

Psychiatric disorders and IBS diagnosis:

Our study reported that higher psychological distress was significantly associated with higher odds of having possible IBS. Previous published studies have shown a strong correlation between IBS and psychiatric disorders like depression and anxiety [37–40]. It has been proven that psychiatric disorders can alter the gut’s motor functioning, sensory threshold and stress susceptibility; the other way around is also true where IBS can impact negative emotions like anxiety and depression [61]. A meta-analysis including case–control and prospective cohort studies concluded that anxiety and depression can double the risk of having IBS in the future [62]. A study conducted in the USA concluded that IBS can alter people’s everyday life, suffer from focusing, encounter difficulties in making arrangements, avoid sex, and leave the house [63].

Furthermore, higher insomnia severity was significantly associated with higher odds of having possible IBS, corroborating the findings of prior studies [26, 43, 44]. A study conducted in order to evaluate the relationship between sleep disruption and gastrointestinal symptoms in women with and without irritable bowel syndrome found data that supported the notion that poor sleep leads to worse gastrointestinal symptoms the next day in women with IBS [64]. A study reported the association between IBS symptoms like abdominal pain and difficulty falling asleep [65]. According to Ali et al. sleep disturbances can be caused by abnormal gastrointestinal immune mechanisms found in gastrointestinal diseases such as IBS [66].

### Moderating effect of smoking

Moreover, a significant moderating effect of smoking on IBS was found in our study. It was shown that in nonsmokers, higher psychological distress and insomnia severity were significantly associated with higher odds of having possible IBS. Many published studies supported our findings with a significant association between IBS and psychological distress and insomnia [26, 37–40, 43, 44]. We speculate a relationship between the gut and the hypothalamic pituitary axis: the corticotropin-releasing hormone, a well-known modulator of stress has been linked with IBS by increasing intestinal permeability [67]. In addition, serotonin, which helps regulate emotions and behaviors such as depression and anxiety, has been implicated in the gut-brain function in functional gastrointestinal disorders such as IBS [67]. Moreover, studies have shown the relationship between the gut microbiota and circadian disturbances leading to insomnia [68].

In smokers, higher eating attitudes scores (more inappropriate eating) were significantly associated with higher odds of having possible IBS. Many studies showed significant association between IBS and eating disorders [45–47]. A study showed that eating disorders were present before the onset of IBS for the vast majority of the participants suggesting that eating disorders are a risk factor for IBS [47]. Another study conducted among adolescents and young individuals reported that IBS participants are more prone to develop eating-associated symptoms that tend to be avoided by restricting the incriminating food [69]. Some types of food were reported to trigger symptoms related to IBS more frequently in participants suffering from IBS than in controls [29], this could be a major factor in the development of eating disorders among IBS patients. We hypothetize that smoking can exacerbate inappropropriate eating attitudes that can lead to higher rates of IBS. In addition*,* the constituents of cigarettes may exacerbate IBS symptoms by promoting mucosal cell death, limiting the cellular regeneration, reducing blood irrigation of the mucosal epithelium in the gastrointestinal tract and impeding with the mucosal immune response [70], leading to higher rates of inappropriate eating disorders*.* Moreover, smoking has been shown to be highly associated with eating disorders according to a meta-analysis [71]. A possible explanation can be related to the fact that smoking can control appetite and lead to weight control, women who smoke primarily to lose weight were found to have significantly higher levels of eating disorders than nonsmokers [72].

The great majority of literature reported an association between IBS and obesity. A prospective cohort study conducted in Norway showed that weight loss can alleviate IBS symptoms [73]. In addition, inflammatory processes have been linked to IBS, these processes can alter the gastrointestinal reflexes [74] and higher levels of inflammatory biomarkers were detected in obese patients with IBS in comparison with controls [75]. However, smokers with higher BMI in our study were significantly associated with lower odds of having possible IBS; this finding can be related to the hypothesized role of cigarette smoking in preventing the development of IBS by reducing the gut mucosal inflammation [76] and slowing the gastrointestinal tract motility [77].

### Clinical implcations

The results of our study may serve as a first step toward implementing better awareness among the Lebanese population about IBS and the Lebanese physicians through helding conferences in schools and universities and distributing brochures in health clinics to increase the rate of diagnosis of IBS; treatment plans, methods of symptom management and appropriate diet program should be developed and spread among health care workers. We should also underline the importance of considering the presence of psychological distress, eating and sleeping disorders among patients diagnosed with IBS.

### Limitations

Some limitations should be taken into consideration while assessing the findings. Although this was a population-based cross-sectional study, the majority of responders were females in their twenties, which might have contributed to the high IBS rate; therefore, the rate of unidentified IBS cannot be generalized to the general population. Moreover, the study was conducted by using self-administered questionnaires only, without adopting neither colonoscopy tests to rule out the presence of intestine damage, or fecal occult blood test to eliminate the presence of blood in stools, or blood tests to eliminate a potential anemia nor a physician assessment of other signs and symptoms, therefore underestimation and exaggeration may occur. A selection bias cannot be eliminated since participants selection was based on the snowball technique, which relied on friends belonging to a certain age group may have affected the age and the socioeconomic class selected. A residual confounding bias might be present since not all factors associated with IBS were included in this study, such as family history of IBS, sexual, emotional or physical abuse and food intolerance.

## Conclusion

In conclusion, our study shed the light on the high rate of unidentified IBS among Lebanese adults, surpassing the highest prevalence of IBS recorded in the database [4]. Our findings found that IBS is a major problem for the Lebanese adult population. It also called attention to the association between IBS and anxiety, depression, eating and sleeping disorders in Lebanon; all these associated factors are preventable and could be managed. We suggest that future researchers conduct more studies about the prevalence of IBS among the general Lebanese population based on a medical assessment done by a physician to get the actual prevalence of IBS.

## Data Availability

Data is not available online but is available upon a logical request to the corresponding author.

## Abbreviations

IBS: Irritable bowel syndrome
EAT-26: Eating Attitudes Test
